# Monoubiquitinated MxIRT1 acts as an iron receptor to determine MxIRT1 vacuole degradation or plasma membrane recycling via endocytosis

**DOI:** 10.1080/15592324.2022.2095141

**Published:** 2022-07-01

**Authors:** Song Tan, Shuang Li, Xiu-Yue Zhang, Yu-Meng Li, Peng Zhang, Li-Ping Yin

**Affiliations:** a School of Pharmacy, Anhui University of Chinese Medicine, Hefei, Anhui, China; b College of Life Science, Capital Normal University, Beijing, China; c Anhui Province Key Laboratory of Research & Development of Chinese Medicine, Hefei, Anhui, China

**Keywords:** Iron, monoubiquitin, MxIRT1, endocytosis, recycle, degradation, receptor

## Abstract

IRON-REGULATED TRANSPORTER 1 (IRT1) is critical for iron uptake in roots, and its exocytosis to the plasma membrane (PM) is regulated by the iron status sensed by the histidine-rich domain (HRM). However, studies on the fate of IRT1 after fusion with PM in response to iron conditions are still limited. In this study, we found that K165 and K196 regulate the monoubiquitination of MxIRT1 (mUb-MxIRT1), which acts as a receptor delivering signals from HRM to downstream effectors such as clathrin to determine the fate of MxIRT1. Iron supply led MxIRT1 in the PM to monoubiquitin-dependent endocytosis which could be inhibited by endocytosis inhibitor TyrA23 or in the double site-directed mutant K165/K196R. Subsequently, the endocytosis pathway to the vacuole was inhibited by vacuolar protease inhibitor Leupeptin in excessive iron conditions and the inability of being able to respond to iron change, indicated by the protein accumulating in the PM, contributed to iron toxicity in K165/K196R transgenic Arabidopsis. With iron availability decreasing again, MxIRT1 could dock close to the PM waiting for to be recycled. Another monoubiquitination site, K26, was necessary for MxIRT1 Endoplasmic Reticulum (ER) export as site-directed mutant K26R lost the ability of PM targeting, and co-localized with the COPII subunit of the coat protein OsSec24. Therefore, after K26-directed ER export and iron-induced PM fusion, mUb-MxIRT1 determines subsequent vacuolar degradation or recycling to the PM via endocytosis for maintaining iron homeostasis.

## Introduction

Iron (Fe) plays indispensable roles in plant growth and development, including respiration, photosynthesis, and antioxidant defenses, and it is, therefore, an essential micronutrient.^18^ As it is present in insoluble complexes (oxidized form, Fe^3+^) in calcareous soils, iron is a limiting factor for plant biomass production and agricultural productivity.^13^ The essential role of iron is highlighted by the severe disorders resulting from its deficiency, such as anemia in mammals or chlorosis and yield loss in plants.^6^ In some areas, the interflow of Fe from the upland slopes, poor water management, industrial discharge, and various anthropogenic activities improves the uptake of Fe^2+^ from the soil. However, Fe^2+^ is readily absorbed by plants and stored in leaves and can result in Fe toxicity.^34^ Fe toxicity causes serious damage to important biomolecules, including lipids, proteins, and DNA, which leads to ferroptosis and induces structural changes in the photosynthetic apparatus, inhibiting carbon metabolism.^19^ Precise control of Fe homeostasis could reduce the risk of progressive damage caused by cellular iron deficiency/excess and its subsequent metabolic costs.

Iron-regulated transporter 1 (IRT1) is the major player in regulating plant iron homeostasis, as attested by severe chlorosis and the lethality of the Arabidopsis *irt1* knockout mutant.^31^ Expression of the IRT1 is mainly controlled by iron at the levels of transcript accumulation.^9^ Upon the onset of low-iron conditions, the basic helix-loop-helix (bHLH) transcription factors increases the expression of FER-LIKE IRON DEFICIENCY-INDUCED TRANSCRIPTION FACTOR (FIT) and forms heterodimers with FIT to drive the transcription of hundreds of genes, including *IRT1* and FERRIC REDUCTION OXIDASE 2 (*FRO2*), thus boosting iron uptake in roots.^8,33^ At the post-translational level, IRT1 could directly sense elevated non-iron metal concentration. Therefore, it has been proposed as a transceptor for metal homeostasis in plants.^7,11^ IRT1 is gradually removed from the plasma membrane (PM) through a monoubiquitin- and clathrin-dependent mechanism via differential ubiquitination on exposure to increasing concentrations of these highly reactive non-iron metals.^3^ In recent years, many studies have uncovered novel factors involved in IRT1 intracellular traffic and facilitated a better understanding of the role of intracellular traffic in the fine balancing of plant metal homeostasis. To limit IRT1-mediated accumulation of metals, direct non-iron metal binding to IRT1 allows the recruitment of the CBL-interacting serine/threonine-protein kinase 23 (CIPK23) and subsequent phosphorylation of IRT1, which in turn creates a docking site and facilitates the recruitment of the E3 Ub ligase IRT1 DEGRADATION FACTOR1 (IDF1). When plants are challenged with increasing concentrations of non-iron metal, IDF1 facilitates IRT1 degradation by converting monoubiquitin into lysine K63-linked polyUb chains.^11^ The PHOSPHATIDYLINOSITOL-3-PHOSPHATE-BINDING PROTEIN (FYVE1) and SORTING NEXIN (SNX) control IRT1 in endosomal compartments back to the plasma membrane based on the decreasing availability in non-iron metal substrates of IRT1.^2,14,23^ However, the knowledge of direct intracellular traffic of IRT1 protein exposure to iron is still limited.

MxIRT1, a high-efficiency Fe^2+^ transporter cloned from *Malus xiaojinensis* roots,^15^ targets to the PM in response to iron supplementation and depends on association with detergent-resistant membranes (DRMs).^29,30^ The histidine-rich domain (HRM) in MxIRT1 acts as an iron sensor to induce the delivery of MxIRT1 vesicles to the PM after directly binding to Fe^2+^ from the environment.^30^ In yeast, MxIRT1 is eventually degraded in the vacuole through autophagy in the presence of excess iron.^16^ PM fusion and vacuolar degradation are key regulatory steps of high-affinity metal transporters that allow plants to switch the metal transport function on and off in response to metal conditions. However, many MxIRT1 regulatory mechanisms remain unknown; for example, it is unclear what initiates MxIRT1 removal from the PM. Additionally, the fate of MxIRT1 after responding to iron concentration changes in the environment sensed by HRM is unknown. Further research is necessary to elucidate these regulations at the post-translational and cellular levels.

In this study, we found three ubiquitination sites (i.e., K26, K165, and K196) involved in MxIRT1 post-translational modification and explored their role in the response of MxIRT1 to iron change for maintaining iron homeostasis. Single lysine site-directed mutants (K26R, K165R, and K196R) and multiple lysine site-directed mutants (K165/K196R and K26/K165/K196R) were constructed and overexpressed in yeast, rice, and Arabidopsis. After detecting protein expression in these materials using immunoblot analysis, immunoprecipitation was used to confirm the ubiquitination modification of MxIRT1 (Ub-MxIRT) and its mutants. Complement analysis, noninvasive micro-test technique (NMT), and inductively coupled plasma mass spectrometry (ICP-MS) further ascertained metal uptake of Ub-MxIRT1. After different iron and inhibitor treatments, the traffic route of Ub-MxIRT1 was further observed by detecting the dynamic change of MxIRT1 localization using confocal microscopy and exploring the cellular distribution of MxIRT1 protein using immunoblot analysis after sucrose density gradient centrifugation. In conclusion, our research investigated the traffic route of MxIRT1 from the PM and the ubiquitination sites in MxIRT1 under different iron conditions to discover the key sites and determine how these sites initiate MxIRT1 removal from the PM. Additionally, we determined the fate of MxIRT1 following the response to iron changes sensed by the HRM for maintaining iron homeostasis.

## Materials and methods

### Yeast strains, transformation, and growth conditions

Yeast strains DEY1453 and DEY1457 were grown in Yeast Extract Peptone Dextrose medium (YPD medium, 2 g/L peptone, 1 g/L yeast extract, and 2 g/L glucose). Transgenic yeast strains were grown in Synthetic Dropout Medium-Uracil (SD-Ura^−^ medium) consisting of SD (6.7 g/L yeast nitrogen base, 0.77 g/L lacking uracil DO supplement) and 0.2 g/L glucose.

The lysine sites-directed mutants were conducted by Polymerase Chain Reaction (PCR) using the PrimeSTAR Max DNA Polymerase and primer pairs K26R-F and K26R-R, K165-F, and K165-R, K196R-F, and K196R-R using pYES2.0-MxIRT1-GFP or PBI221-MxIRT1-GFP as template. The primer sequences were listed in Table S1. The plasmid constructs were transformed into yeast DEY1453 or DEY1457 using the highly efficient Li-acetate transformation method and the transformed yeasts were grown on SD-Ura^−^medium.

In the complementation and protein detection assays, glucose was replaced by galactose to drive the protein expression by the galactose (GAL1) promoter present in the pYES2.0 vector. Yeast cell was diluted to optical density of OD_600_ equal to 0.1, 0.01, 0.001, and 0.0001. Thereafter, 4 μL of each cell suspension was dropped on SD medium lacking uracil and supplemented with 30 μmol L^−1^ BPDS (batho-phenanthroline disulfonic acid) for the Fe-limited treatments (-Fe), 50 μmol L^−1^ EDTA-Fe for Fe-sufficient treatments (+Fe), or 1 mmol L^−1^ EDTA-Fe for Fe-excessive treatments (++Fe). The plates were incubated at 28°C for 3 d.

### Arabidopsis and rice protoplasts preparation, transformation, and growth conditions

Mutant *irt1* seeds were sterilized, placed in the dark at 4°C for 2 d, and sown on 1/2 Murashige and Skoog (MS) medium plates supplemented with 2% sucrose and 1% agar, pH 5.8. For Arabidopsis transformation, all the constructs were introduced into *A. tumefaciens* strain GV3101 by heat shock. The agrobacterium clones were then used to transform the Arabidopsis through a floral dipping method.^36^ Transgenic plants were selected on 1/2 MS agar plates with corresponding antibiotic (20 mg/L hygromycin) and homozygous T3 lines were used for further analysis. Transgenic plants were grown on plates supplemented with hygromycin (20 g/mL).

Wild type rice seeds were sown on MS medium. For rice protoplast transformation, protoplasts were resuspended in W5 solution (154 mM NaCl, 5 mM KCl, 125 mM CaCl_2_, 2 mM MES, pH 5.7) and centrifuged at 1,500 rpm for 5 min to carefully removed suspension. The protoplasts were then resuspended in MMG solution (15 mM MgCl_2_, 4 mM MES, 0.6 M mannitol, pH 5.7) and add plasmid DNA to protoplast, and mix gently. Thereafter, PEG solution (40% PEG4000, 0.6 M mannitol, 100 mM CaCl_2_) was added and complete mixed by gently tapping the tube. The transfection mixture was incubated at room temperature for up to 15 min. The transfection mixture was then diluted with W5 solution at room temperature and mixed well by gently rocking or inverting the tube to stop the transfection process. The mixture was then centrifuged at 1,500 rpm for 5 min to removed suspension, and protoplasts were resuspended gently with incubation solution (0.6 mM mannitol, 4 mM MES, 4 mM KCl, pH 5.7) in a plate, incubated at room temperature for 12–18 h, and last used for detecting protein location and Cd^2+^ flux.

### Observation of subcellular localization of protein in yeast and rice protoplasts

Confocal microscopy was performed on an LSM 780 (Zeiss). For green fluorescent protein (GFP) visualization, excitation at 488 nm and detection between 505 and 545 nm were used. For FM4-64, excitation at 561 nm and detection from 575 to 615 nm were used. Pinholes for both channels were set to 1 Airy Unit resulting in optical slices of 0.8 mm. Images were recorded in a 1024 pixel format. Colocalization analysis was performed on 8-bit gray-scale image pairs, representing the GFP and FM4-64 channels.

### Cd^2+^ flux measured by noninvasive micro-test technique (NMT) in yeast and rice protoplasts

The net Cd^2+^ flux in yeast and rice protoplasts were measured using a noninvasive micro-test technology (NMT®; Xuyue Science and Technology Co., Ltd.). The yeast and protoplasts were measured in the testing solution, which contained 0.05 mM CdCl_2_. The microelectrode was then filled with a column of a selective liquid ion-exchanger cocktail (LIX) imparting ion-selectivity with the column length of Cd^2+^ at 15–20 μm. Cd^2+^ ion flux was calculated by Fick’s law of diffusion: J = -D (dc/dr), where J represents the ion flux (10^−12^ mol cm^−2^ s^−1^), dc is the ion concentration difference (10^−3^ mol/L), dr is the distance between two measured points (μm), and D is the ion diffusion constant in a particular solution and temperature (cm^2^/s). Probing, data recording, digital image acquisition and calibrations were performed using the ASET software. All the data were statistically analyzed.

### Measurement of metal content in yeast and Arabidopsis seedlings

Fe and Cd concentrations in yeast strains, and Fe concentration in Arabidopsis seedlings were measured by inductively coupled plasma mass spectrometry (ICP-MS).^26^ The induced yeast cell and Arabidopsis seedlings were cleaned, divided into three groups and dried before being weighed, it was then treated with nitric acid and measured by ICP-MS.

### Protein extraction, immunoblot analysis, and immunoprecipitation

Total Arabidopsis protein was extracted from 14-day-old Arabidopsis seedling. Collected seedlings were ground in liquid nitrogen and resuspended in Radio Immunoprecipitation Assay (RIPA) buffer (50 mM Tris-HCl pH7.5, 150 mM NaCl, 0.5% sodium deoxycholate, 1% Nonidet P-40, 0.1% SDS, and protease inhibitor cocktail mixture) for 10 min on ice. After centrifugation at 15, 000 × *g* for 15 min at 4°C, total Arabidopsis protein was collected, and prepared for immunoblot analysis. Total rice protein was extracted from MxIRT1 T3 transgenic rice seedlings with MG132 (Sigma Aldrich), Leupeptin (Sigma Aldrich), TyrA23 (Sigma Aldrich), CHX (Sigma Aldrich) treatment. Rice seedlings were ground in liquid nitrogen and resuspended in RIPA buffer for 10 min on ice. After centrifugation at 15, 000 × *g* for 15 min at 4°C, total rice protein was collected, and prepared for immunoblot analysis. The yeast cells lysed with snailase at 37°C for 1 h were collected, and then resuspended in RIPA buffer for 10 min on ice. After centrifugation at 15, 000 × *g* for 15 min at 4°C, total yeast protein was collected, and prepared for immunoblot analysis. Proteins were separated by SDS-PAGE and transferred to a PVDF (polyvinylidene difluoride) membrane by electroblotting. Immunoblot analysiss were processed using GFP antibody (santa cruz biotechnology: sc-9996), Pma1 antibody (santa cruz biotechnology: sc-57978), OsHSP80 antibody (Beijing protein innovation), P4D1 antibody (santa cruz biotechnology: sc-8017), Tubulin antibody (Beijing Protein Innovation), and GAPDH antibody (Beijing Protein Innovation).

About 300 μL total proteins were obtained from the procedures described above and supernatant was incubated with 3 μL anti-GFP antibody for 4 h at 4°C on a rotating wheel. The samples were then incubated with 20 μL Protein G Sepharose 4 Fast Flow (GE, 17–0618-01) for 3 h at 4°C on a rotating wheel. Before the incubation, beads were washed with 500 μL PBS three times. The GFP-IgG complexes were eluted from the beads by adding 48 μL RIPA, then with 10 μL 5× SDS loading buffer and heated at 95°C for 6 min. The acquired proteins were then used for immunoblot analysis.

## Statistical analysis

All experiments were performed at least in triplicate. One-way ANOVA was used to compare the quantitative difference between the different samples. Values of P < .05 were considered statistically significant.

## Results

### MxIRT1 is targeted to different destinations in response to different iron treatments

**Figure 1.**
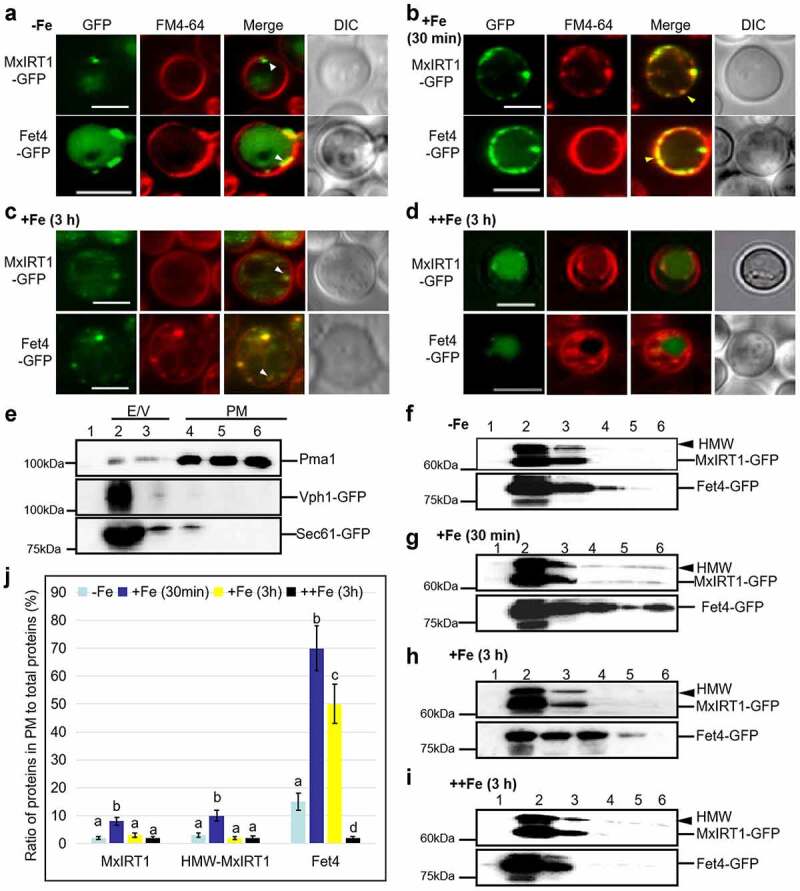
**The response of MxIRT1 and Fet4 to iron change in transgenic yeast**. The subcellular localization of MxIRT1-GFP and Fet4-GFP in transformed DEY1453 (*fet3fet4*) yeast cultured in iron-deficient conditions (30 µM BPDS, – Fe) (a), iron-sufficient condition (50 µM EDTA-Fe, +Fe) for 30 min (b) and for 3 h (c), and iron-excessive condition (1 mM EDTA-Fe, ++Fe) for 3 h (d) after overnight iron-deficient treatment. GFP, green fluorescence protein indicates the location of fusion proteins; FM4-64, red fluorescence as a position indicator of the cell plasma membrane; Merge, images overlaid with both green and red fluorescence; DIC, differential interference contrast. White arrows indicate the intracellular distribution of MxIRT1 or Fet4. Yellow arrows indicate the accumulation of MxIRT1 or Fet4 in the plasma membrane. The fluorescence was detected by confocal microscopy after FM4-64 staining for 5 min. Scale bars for all panels = 5 µm. **E**. Immunoblot analysis of yeast cell lysates fractions from Vph1-GFP and Sec61p-GFP transgenic DEY1453 through Optiprep density gradient centrifugation using anti-Pma1 and anti-GFP antibody. Plasma membrane (PM, fractions 4–6) could be efficiently separated from ER and vacuole (E/V, fractions 2–3). Immunoblot analysis of Fet4-GFP and MxIRT1-GFP levels at the PM fractions or E/V fractions in transformed yeast cultured in – Fe (30 µM BPDS) (f), +Fe (50 µM EDTA-Fe) for 30 min (g) and 3 h (h), and ++Fe (1 mM EDTA-Fe) for 3 h (i) after −Fe overnight treatment using an anti-GFP antibody. Arrows indicate high molecular weight of MxIRT1 (HMW-MxIRT1), and lines indicate Fet4-GFP or MxIRT1-GFP. **J**. The ratio of MxIRT1, HMW-MxIRT1, and Fet4 in the PM to total proteins under the different iron conditions from F, G, H and I. Every independent measurement contains three replicates. Bars, mean ± standard errors (n = 3). Different letters indicate significant differences (P < .05) as determined by ANOVA.

In our previous work, we found that iron addition stimulated the delivery of MxIRT1 vesicles to the PM in plants under iron deficiency.^30,32^ Excessive iron leads to MxIRT1 protein in transgenic yeast cells being degraded in the vacuole through autophagy.^16^ However, the response of MxIRT1 to different iron statuses between targeting the PM after iron addition and the vacuolar degradation in the presence of excessive iron is still unknown. To investigate the dynamic change of MxIRT1 localization in response to iron, we treated MxIRT1 transgenic yeast with different iron concentrations for different periods. Under iron deficiency conditions, MxIRT1 mainly displayed in vesicles close to PM (Figure 1a, white arrow). Fet4, an endogenous iron transporter in yeast,^10^ also mainly appeared in vesicles close to PM under iron-deficient conditions (Figure 1a, white arrow). These two iron transporters were induced by iron deficiency and displayed low PM localization (Fig S1), and accumulated close to the PM for potential PM fusion. After iron supplementation (+Fe) in a short time (30 min), some of the MxIRT1 vesicles were targeted to the PM (Figure 1b, yellow arrow, Fig S1). Similarly, most Fet4 vesicles were targeted to the PM for iron absorption after iron supplement in a short time (30 min) (Figure 1b, yellow arrow, Fig S1). This result is similar to a previous report that MxIRT1 vesicles fuse with the PM after iron supply.^32^ Even though Fet4, the endogenous iron transporter, could stay in the PM longer than MxIRT1 (Figure 1b, g, h, j), most of MxIRT1 and Fet4 disappeared from the PM and were observed as vesicles close to PM after prolonged iron treatment (about 3 h) (Figure 1c, i, j, Fig S1). We speculate that MxIRT1 may be removed from the PM under abundant iron conditions to prevent iron toxicity. Our following experiment confirmed this hypothesis as both MxIRT1 and Fet4 accumulated in the vacuole and disappeared from the PM in the presence of excessive iron (++Fe) (Figure 1d).

We then further explored the distribution of MxIRT1 protein in yeast in response to different iron conditions using immunoblot analysis after sucrose density gradient centrifugation. The ER/vacuole (E/V) and PM fractions could be separately purified from transformed yeast and evaluated by immunoblot analysis as previously reported.^35^ Pma1, one of the most enriched yeast plasma membrane proteins, could be used as a PM marker.^12^ Vph1 is a vacuole-localized V-type H^+^-ATPase and has been used as a vacuole marker.^17^ Sec61, a subunit of the Sec61 complex consisting of protein transport channels in the endoplasmic reticulum membrane, has been used as an ER marker.^4^ We fused GFP protein to Vph1 and Sec61 and detected these protein distributions using a GFP antibody. After centrifugation and protein blotting using different markers from transgenic yeast, Pma1 was mainly distributed in fractions 4–6, defining these as the PM fractions (PM). Vph1 and Sec61 were mainly detected in fractions 2–3, defining these as the ER or vacuole fraction (E/V) (Figure 1e). Under iron-deficient conditions, MxIRT1 and Fet4 were mainly distributed in the E/V fraction (Figure 1f); thus, iron transporter vesicles might be confined intracellularly, waiting for a further signal to fuse with the PM. This was similar to the results of MxIRT1 localization under iron-deficient conditions (Figure 1a). After iron supplementation, the percentages of MxIRT1, HMW-MxIRT1, and Fet4 in the PM increased markedly (Figure 1g, j), indicating that these proteins were targeted to the PM for iron absorption. After prolonged (about 3 h, Figure 1h) and excessive iron (Figure 1i) treatment, the protein bands of MxIRT1 and Fet4 from the PM fractions disappeared (Figure 1j). These results indicate that both exogenous MxIRT1 and endogenous Fet4 vesicles in yeast accumulated closed to the PM under iron-deficient conditions and could be targeted to the PM in response to iron addition. Thereafter, MxIRT1 may be removed from the PM under prolonged abundant iron conditions. Whether the same dynamic change in MxIRT1 localization in response to iron change happens in plants and how MxIRT1 disappears from the PM under abundant iron conditions are unknown.

### MxIRT1 in the PM maintains iron homeostasis under different iron statuses in plants

**Figure 2.**
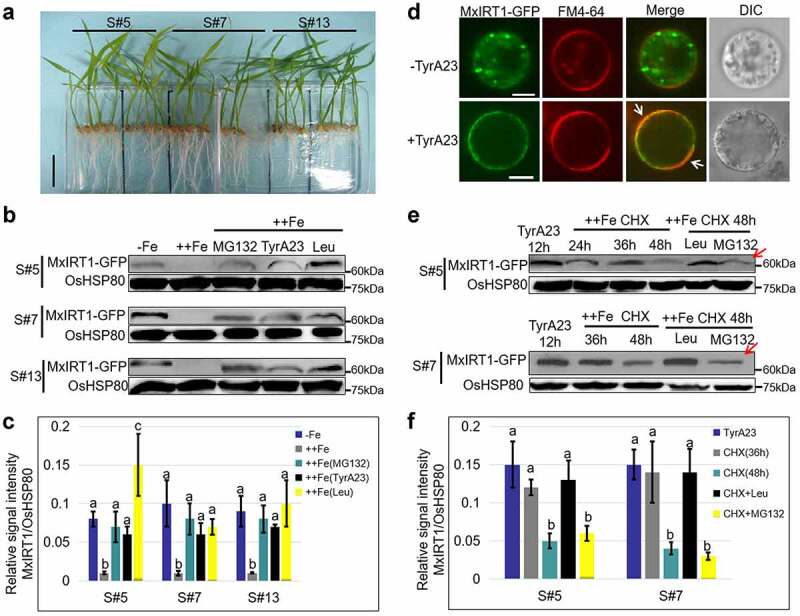
**The response of MxIRT1 to iron change in T3 MxIRT1 transgenic rice seedlings**. Representative appearance of T3 MxIRT1 transgenic rice seedlings used for the following experiments. S (sense) represented MxIRT1 overexpression lines, Scale bars 5 cm. **B**. The expression of MxIRT1 responding to iron change in transgenic rice seedlings with or without Fe and inhibitors. Transgenic rice sense lines (S#5, 7, and 13) were grown for 3 weeks on MS growth medium (100 µM Fe, +Fe), and for another 6 d under iron-deficient growth medium (0 µM Fe, -Fe). The seedlings were then treated with 12 h excessive iron (1 mM Fe, ++Fe) or 12 h ++Fe with 100 µM Leupeptin, 100 µM TyrA23 or 40 µM MG132. Total protein was prepared for immunoblot analysis with an anti-GFP antibody. OsHSP80 was used to verify equal loading using anti-OsHSP80 antibody. **C**. The histogram represents the quantification of the MxIRT1 bands from B by Image J. MxIRT1 protein levels are expressed as a ratio of MxIRT1 to OsHSP80. Every independent experiment contains three replicates. Bars, mean ± standard errors (n = 3). Different letters indicate significant differences (P < .05) as determined by ANOVA. **D**. The localization of MxIRT1-GFP in transgenic rice protoplasts with (+TyrA23) or without (-TyrA23) TyrA23 application. White arrows indicate the accumulation of MxIRT1 in the plasma membrane. GFP, green fluorescence indicated the location of fusion protein; FM4-64, red fluorescence as a position indicator of the cell plasma membrane; Merge, images overlaid with both green and red fluorescence; DIC, differential interference contrast. Fluorescence was observed after FM4-64 staining for 5 min. Scale bars 10 µm. **E**. A time course analysis of MxIRT1-GFP level in transgenic sense lines (S#5 up, S#7 down) under excessive iron conditions (1 mM Fe, ++Fe) in the presence of CHX after 12 h of 100 µM TyrA23 treatment, and the level of MxIRT1-GFP under ++Fe for 48 h in the presence of CHX and Leupeptin or MG132 following 12 h of 100 µM TyrA23 treatment. Red arrows indicate the disappearance of MxIRT1 even after MG132 treatment. Total protein was prepared for immunoblot analysis with an anti-GFP antibody. OsHSP80 was used to verify equal loading using an anti-OsHSP80 antibody. **F**. The histogram represents the quantification of the MxIRT1 bands from E by Image J. MxIRT1 protein levels are expressed as a ratio of MxIRT1 to OsHSP80. Every independent experiment contains three replicates. Bars, mean ± standard errors (n = 3). Different letters indicate significant differences (P < .05) as determined by ANOVA.

To analyze the response of MxIRT1 to the iron conditions in plants, we used the T3 MxIRT1 transgenic rice lines S5, S7 and S13 constructed previously,^26^ and treated them with different iron concentrations (Figure 2a). Under iron deficiency, the expression of MxIRT1 protein was induced and could be detected in transgenic rice lines S5, S7, and S13 (Figure 2b, -Fe). MxIRT1 has also been detected under normal (+Fe) conditions in our previous work.^26^ However, MxIRT1 decreased remarkably and was hard to detect in the presence of excessive iron (++Fe) (Figure 2b, c). This phenomenon was observed when it was reported that AtIRT1 was gradually removed from the PM through monoubiquitin- and clathrin-dependent endocytosis and targeted to the vacuole for turnover upon exposure to increasing concentrations of non-iron metals to avoid non-iron metal toxicity.^3,11^ The IRT1 from the sorting endosome after endocytosis could retrograde transport to early endosome for PM fusion regulated by FYVE1 and SNX with decreasing metal availability.^2,14^ Therefore, there could be two possible routes for the MxIRT1 disappearance from the plasma membrane in plants: one is the endocytosis of MxIRT1 followed by docking close to PM for recycling to the PM under decreasing iron availability, and the other is targeting to the vacuole through endocytosis for turnover under excessive iron condition.

To investigate the route of MxIRT1 disappearance from the PM, we treated transgenic rice seedlings with different inhibitors. The first was Leupeptin, a vacuolar protease inhibitor. After treatment with Leupeptin, MxIRT1 was accumulated in transgenic rice lines S5, S7, and S13 under excessive iron conditions (Figure 2b, c), indicating the involvement of vacuolar protease in MxIRT1 degradation. The second is the 26S proteasome inhibitor MG132. 26S proteasome, another degradation way, has been reported to play a critical role in iron homeostasis by regulating the turnover of transcriptional activator FIT and OsIDEF1.^24,27^ Impairment of the 26S proteasome by MG132 treatment resulted in accumulation of MxIRT1 in transgenic rice lines S5, S7, and S13 under excessive iron conditions (Figure 2b, c), indicating the involvement of the proteasome in MxIRT1 degradation. Treatment with the two inhibitors demonstrated that both the vacuole and proteasome are involved in the turnover of MxIRT1 in the presence of excessive iron in plants. The third is TyrA23, an inhibitor of cargo sorting into clathrin-coated vesicles,^1^ which were used to treat transgenic rice seedlings and rice protoplasts for exploring the disappearance route of MxIRT1 removing from PM. Blocking clathrin-mediated endocytosis with TyrA23 resulted in the accumulation of MxIRT1 in the plasma membrane at the cellular level (Figure 2d, white arrow), demonstrating that MxIRT1 went through clathrin-dependent endocytosis from the PM. At the protein level, TyrA23 treatment could maintain the stability of MxIRT1 protein in the ++Fe condition in the S5, S7, and S13 lines (Figure 2b, c). Considering the accumulation of MxIRT1 protein after endocytosis inhibitor TyrA23 treatment in the ++Fe condition (Figure 2b, c) and docking of the MxIRT1 vesicle close to PM from iron abundance to deficiency (Figure 1a), the endocytic MxIRT1 from PM gets into disappearance route including preparing for recycling to PM as vesicle and targeting to degradation.

We further researched the intracellular route after MxIRT1 endocytosis from the PM in plants. With a 12 h TyrA23 treatment of rice seedlings, MxIRT1 was accumulated in the PM, which could mimic the MxIRT1 in the PM, performing its iron-absorbing function and waiting for further signals (Figure 2d). After the TyrA23 treatment, transgenic rice seedlings were treated with a protein synthesis inhibitor (cycloheximide, CHX) under extreme iron conditions (1 mM Fe, ++Fe) for 24, 36 and 48 h to inhibit MxIRT1 synthesis (Figure 2e). Combining the TyrA23 with CHX treatments will prevent the de novo synthesis of MxIRT1, and the protein bands will reflect the relationship between the amount of protein in the PM after inhibition of endocytosis and environmental conditions. The MxIRT1 in the PM could still be degraded in response to excessive iron as MxIRT1 bands decreased over time (Figure 2e, f). To investigate where endocytic MxIRT1 is located after 48 h, rice seedlings were treated with Leupeptin or MG132 after 48 h of ++Fe, CHX, and TyrA23 treatment. The results showed that Leupeptin treatment could inhibit the decreasing of MxIRT1 after TyrA23 and CHX treatment led to the accumulation of MxIRT1, while MG132 could not inhibit the disappearance of MxIRT1 (Figure 2e, red arrows; Figure 2f). The result confirmed the vacuole targeted degradation of endocytic MxIRT1 from the PM in excessive iron conditions. These results indicate that MxIRT1 in the PM maintains iron homeostasis through clathrin-mediated endocytosis under abundant iron conditions and is recycled to the PM when iron becomes available after iron deficiency (-Fe) or is targeted to the vacuole for degradation in excessive iron conditions in plants. Furthermore, whether the response of MxIRT1 in the PM to iron change is regulated by ubiquitination, a post-translational regulation has been found in AtIRT1 in response to non-iron metal change,^3^ drawing our attention.

### K26, K165, and K196 are the critical sites for MxIRT1 ubiquitination

**Figure 3.**
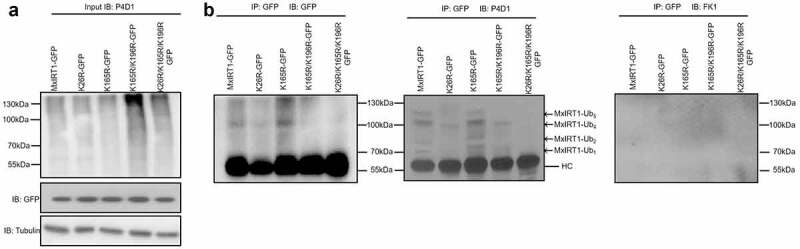
**Immunoprecipitation analysis of the ubiquitination of MxIRT1 and MxIRT1 lysine site-directed mutants in yeast. A**. Immunoblot analysis of yeast cell lysates from MxIRT1 and MxIRT1 lysine site-directed transgenic yeast mutants by the anti-P4D1 (IB: P4D1) and anti-GFP (IB: GFP) antibodies and it was then used as a loading control for the input of the immunoprecipitation. IB, immunoblotting. Equal protein loading was confirmed by using the anti-tubulin antibody (IB: Tubulin). **B**. Total proteins from MxIRT1 and MxIRT1 lysine site-directed mutants transgenic yeast were immunoprecipitated by anti-GFP antibody (IP: GFP) and detected by anti-GFP antibody (IB: GFP), anti-ub antibody (IB: P4D1), or anti-Polyub antibody (IB: FK1). IB, immunoblotting; IP, immunoprecipitation. The heavy chain (HC) of the GFP antibody was indicated. MxIRT1-Ub1 to MxIRT1-Ub5 indicates the numbers of ubiquitination linked to MxIRT1 or MxIRT1 mutants.

Ubiquitination of plasma membrane proteins, including AtIRT1 from Arabidopsis, is a prerequisite for endocytosis and degradation in yeast and mammals.^3,5^ To analyze the mechanism of MxIRT1 endocytosis from the PM in response to iron changes and the involvement of ubiquitination, we predicted the ubiquitination sites in MxIRT1 using Ubpredict.^21^ Three lysines in MxIRT1, i.e., K26, K165, and K196, showed the highest possibility for ubiquitination. K26 was in the uncleaved signal peptide (SP) in MxIRT1.^35^ K165 and K196 were in the large loop between transmembrane domain III and transmembrane domain IV of MxIRT1.^28^ AtIRT1 has two lysine ubiquitination sites (i.e., K154 and K179) in the large loop, which is involved in monoubiquitination and endocytosis.^3^ Thus, we constructed the single lysine site-directed mutants K26R, K165R, and K196R, the double lysine site-directed mutant K165/K196R, and the triple lysine site-directed mutant K26/K165/K196R and overexpressed these proteins in yeast to explore their role in MxIRT1 ubiquitination and function. Immunoblot analysis was used to confirm the protein expression of MxIRT1-GFP, K26R–GFP, K165R-GFP, K165R/K196R-GFP, and K26R/K165R/K196R-GFP in transgenic yeast using a GFP antibody (Figure 3a). MxIRT1 and the mutants were immunoprecipitated from yeast transformants and detected using an anti-GFP antibody, the anti-ubiquitin antibody P4D1, and the anti-polyubiquitin antibody FK1 (Figure 3b). P4D1 is an antibody that recognizes mono-ubiquitin and polyubiquitin chains, while FK1 can only recognize polyubiquitin chains.^16^ Immunoblot analysis of GFP immunoprecipitates from transformants with anti-GFP antibody and anti-ubiquitin antibody (P4D1) revealed high molecular-weight bands in the range where the MxIRT1–ubiquitins conjugates were predicted (Figure 3b). Compared to MxIRT1 and K165R, the ubiquitin conjugates of K26R and K165/K196R were decreased, and K26/K165/K196R eliminated the ubiquitination. This result indicated that K26, K165, and K196 are involved in MxIRT1 ubiquitination. As Ub-MxIRT1 has not been detected using an antibody specific for polyubiquitination (FK1) (Figure 3b) and MxIRT1 is labeled with multi-mono-ubiquitins,^16^ K26, K165, and K196 are the lysine sites regulating the monoubiquitination of MxIRT1.

### K26 is the site regulating ER exporting

**Figure 4.**
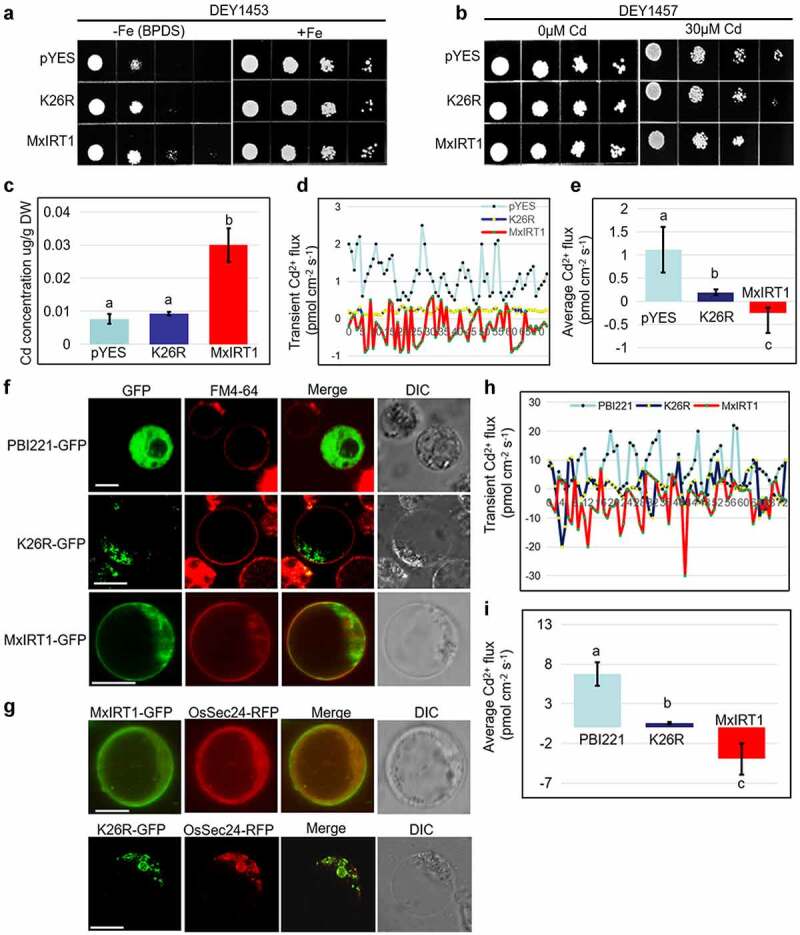
**Functional and localization analysis of MxIRT1 K26 point mutant. A**. Complement analysis of Fe uptake in K26 point mutant. Yeast mutant strain DEY1453 (*fet3/fet4*) containing pYES, K26R, and MxIRT1 was diluted to an optical density of OD600 equal to 0.1, 0.01, 0.001 and 0.0001 and dotted (4 µl) on SD-Ura medium containing 30 µM BPDS (a ferrous chelate, -Fe) or 50 µM EDTA-Fe (+Fe) condition at 28°C for 3 d. **B**. Complement analysis of Cd uptake in K26 point mutant. Yeast mutant strain DEY1457 containing pYES, K26R, and MxIRT1 are grown on SD-Ura medium containing 0 µM CdCl_2_ or 30 µM CdCl_2_ condition. **C**. Cd content in transgenic yeast is measured by using inductively coupled plasma mass spectrometry (ICP-MS). Every independent measurement contains three replicates. Bars, mean ± standard errors (n = 3). Different letters indicate significant differences (P < .05) as determined by ANOVA. DW, dry weight. **D**. Presentation of transient Cd^2+^ flux by NMT in the transgenic yeast in 0.05 mM CdCl_2_. **E**. Average Cd^2+^ flux by NMT in the transgenic yeast. Every independent measurement contains >80 replicates. Bars, mean ± SD. Different letters indicate significant differences (P < .05) as determined by ANOVA. **F**. Subcellular localization of PBI221, K26R, MxIRT1 in rice protoplasts. GFP, green fluorescence protein indicates the location of fusion proteins; FM4-64, cell plasma membrane dye; Merge, images overlap with both green fluorescence and red fluorescence; DIC, differential interference contrast. Fluorescence was observed 5 min after adding FM4-64. Scale bar, 10 µm. **G**. Co-localization of MxIRT1-GFP or K26R-GFP with the subunit of the COPII coat OsSec24-GFP in rice protoplasts. Scale bar, 10 µm. **H**. Presentation of transient Cd^2+^ flux by NMT in the transgenic rice protoplasts in 0.05 mM CdCl_2_. **I**. Average Cd^2+^ flux by NMT in the transgenic rice protoplasts. Every independent measurement contains >80 replicates. Bars, mean ± SD. Different letters indicate significant differences (P < .05) as determined by ANOVA.

To research the function of the lysines regulating monoubiquitination of MxIRT1, we analyzed the metal transport ability and subcellular localization of site-directed mutants in yeast strains and rice protoplasts. Compared to MxIRT1, K26R lost the ability to rescue the growth of yeast mutant DEY1453 (Figure 4a). The K26R transgenic yeast displayed higher tolerance to high cadmium concentration (Figure 4b) because of less cadmium uptake than MxIRT1 transgenic yeast (Figure 4c). The noninvasive micro-test technique (NMT), a technique wildly used to test the metal flux in cell membranes, was also used to detect the metal transport ability of transgenic yeasts.^29^ The value of transient and average Cd^2+^ flux in the MxIRT1 transgenic yeast surface was negative, indicating the MxIRT1 transport of Cd from the medium into the cells. In contrast, the value of the transient and average Cd^2+^ flux in the K26R transgenic yeast surface was positive, indicating that K26R exported Cd from the cell and lost the ability to transport Cd^2+^ from the medium into the cells (Figure 4d, e). These results illustrated that K26R lost the ability to uptake metals.

Compared to MxIRT1 vesicle fusion with PM in rice protoplasts, most K26R could not target to the PM (Fig S2) and was mainly distributed intracellularly (Figure 4f). As the K26 site is the site in the signal peptide, and the uncleaved signal peptide directs MxIRT1 into the ER for the PM secretory pathway,^35^ K26 might be involved in the regulating function of the ER exporting of the MxIRT1 signal peptide. Sec24, a subunit of the COPII coat protein, is required for protein exit from the ER and has been used as an ER marker.^29^ As expected, K26R showed co-localization with OsSec24 in the ER of rice protoplasts (Figure 4g) and was mainly localized in ER (Fig S2). Transient Cd^2+^ flux and average Cd^2+^ flux in rice protoplasts showed that MxIRT1 had a negative value in transient Cd^2+^ flux, indicating that MxIRT1 transported Cd^2+^ from the medium into the cells. In contrast, K26R had a positive value in transient Cd^2+^ flux, indicating that K26R exported Cd^2+^ from the cell and lost the ability to transport Cd^2+^ into the cells (Figure 4h, i). The function and localization results indicate that K26 is the key amino acid regulating MxIRT1 ER exporting through monoubiquitination.

### K165 and K196 regulated monoubiquitination governs MxIRT1 iron uptake and endocytosis

**Figure 5.**
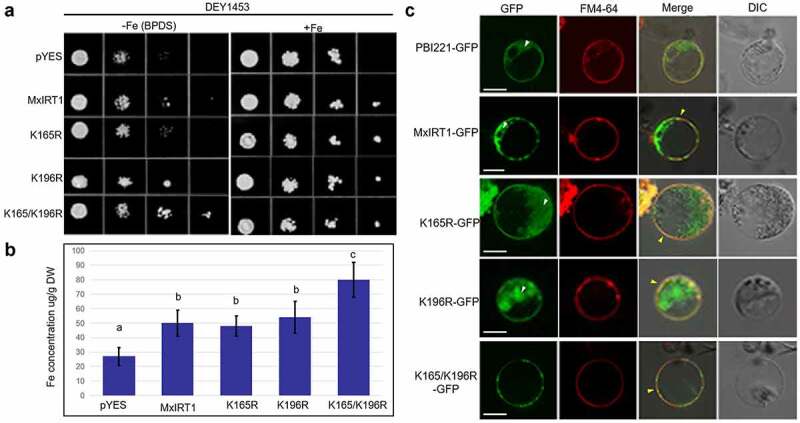
**Functional and localization analysis of MxIRT1 K165 and K196 point mutants. A**. Complement analysis of Fe uptake in K165 and K196 point mutants. Yeast mutant strain DEY1453 containing pYES, MxIRT1, K165R, K196R, K165R/K196R are diluted to an optical density of OD_600_ equal to 0.1, 0.01, 0.001 and 0.0001 and dotted (4 µl) on SD-Ura medium containing +Fe (50 µM EDTA-Fe) or -Fe (30 µM BPDS, a ferrous chelate) condition at 28°C for 3 d. **B**. Iron content in transgenic yeast grown in +Fe condition is measured using inductively coupled plasma mass spectrometry (ICP-MS). Every independent measurement contains three replicates. Bars, mean ± standard errors (n = 3). Different letters indicate significant differences (P < .05) as determined by ANOVA. DW, dry weight. **C**. Subcellular localization of PBI221, MxIRT1, K165R, K196R, K165R/K196R in rice protoplasts. GFP, green fluorescence protein indicates the location of fusion proteins; FM4-64, cell plasma membrane dye; Merge, images overlap with both green fluorescence and red fluorescence; DIC, differential interference contrast. Fluorescence was observed 30 min after adding FM4-64. Scale bar, 10 µm. White arrows indicate the intracellular distribution of MxIRT1. Yellow arrows indicate the accumulation of MxIRT1 in the plasma membrane.

After confirming that K26 is the ubiquitination site of MxIRT1 and directs ER exporting for PM secretory pathway, we analyzed other ubiquitin sites. As lysine site-directed mutant K165/K196R could decrease the Ub-MxIRT1 (Figure 3), we explored the role of K165/K196 mediated monoubiqutination in MxIRT1 function through analyzing the metal transport ability of these site-directed mutants. Complementary functional analysis showed that MxIRT1, single lysine site-directed mutants K165R and K196R, and double lysine site-directed mutant K165/K196R could restore the growth of the yeast *fet3fet4* mutant DEY1453, which is defective in both high- and low-affinity iron uptake (Figure 5a). Simultaneously, the K165/K196R transgenic strain grew better than the MxIRT1, K165R, and K196A transgenic strains under iron deficiency (Figure 5a). Additionally, the iron concentration in K165/K196R transgenic strain was higher than that in the other transgenic strains measured using inductively coupled plasma mass spectrometry (ICP-MS) (Figure 5b). Thus, impairment of the K165 and K196 mediated monoubiquitination could improve the iron uptake ability of MxIRT1 in yeast.

To explore the function of K165 and K196 mediated monoubiquitination on MxIRT1 localization, we detected the localization of MxIRT1 and MxIRT1 mutants after adding a GFP tag sequence in rice protoplasts using laser scanning confocal microscopy (Figure 5c). Free GFP (PBI221-GFP) showed a wide dispersal pattern throughout the cytosol (Figure 5c, white arrow). Meanwhile, MxIRT1-GFP and the single lysine site-directed mutants K165R and K196R were distributed in the PM, as indicated by the presence of partial co-localization with FM4-64 (Figure 5c, yellow arrows). In addition to the PM localization of MxIRT1, K165R, and K196R, the green signal was observed intracellularly (Figure 5c, white arrow). However, the double lysine site-directed mutant K165/K196R was not observed intracellularly, but accumulated in the PM (Figure 5c, yellow arrow). As MxIRT1 was removed from the PM through endocytosis (Figure 2d), displacement of both K165 and K196 might impair the endocytosis of MxIRT1, leading to accumulation of MxIRT1 in the PM following an improvement in iron uptake ability. The function and localization results indicated that K165 and K196 in the large loop are the key amino acids that regulated MxIRT1 endocytosis from the PM through monoubiquitination. As MxIRT1 has been identified as an iron sensor by directly binding to Fe^2+^ through HRM in the large loop^30^ and the monoubiquitination sites K165 and K196 are in the same large loop with HRM, mUb-MxIRT1 may act as a receptor involved in the iron information transfer process from the MxIRT1-Fe^2+^ complex to downstream effectors. Under abundant iron conditions, the increasing MxIRT1-Fe^2+^ complex at PM leads to mUb-MxIRT1, which recruits clathrin for endocytosis. Under excessive iron conditions, the toxic MxIRT1-Fe^2+^ complex in the cell leads to mUb-MxIRT1 recruiting ESCRT complex (ENDOSOMAL SORTING COMPLEX REQUIRED FOR TRANSPORT) for vacuolar targeting. With decreasing iron availability, the released MxIRT1 from MxIRT1-Fe^2+^ complex leads to Ub-MxIRT1 recruiting FYVE1 or SNX1 for recycling to PM.

### K165 and K196 regulated monoubiquitination is necessary for maintaining iron homeostasis in Arabidopsis

**Figure 6.**
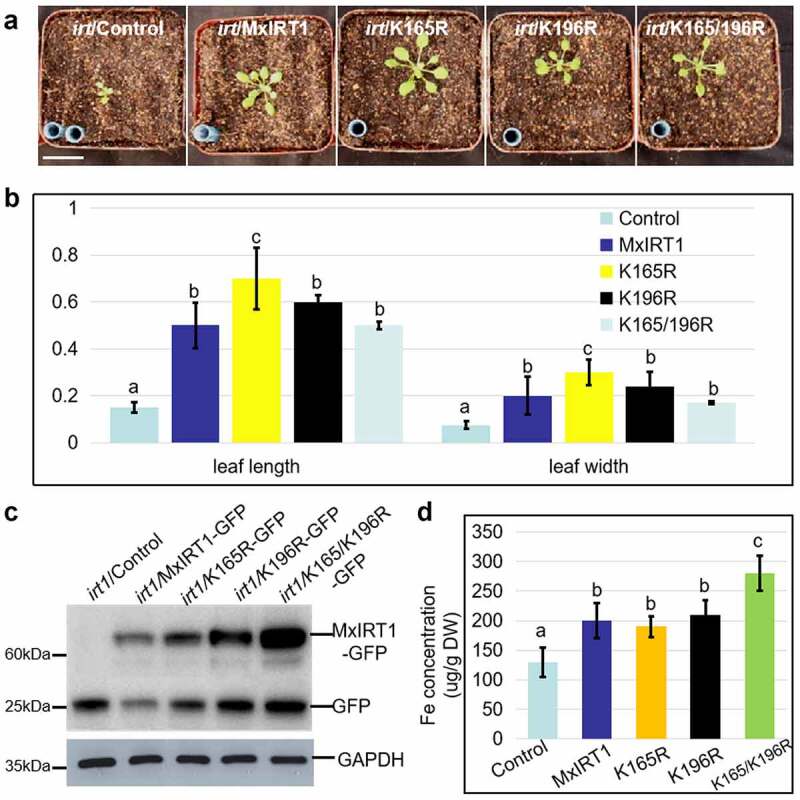
**Phenotypic and iron uptake analysis of MxIRT1 lysine site-directed mutagenesis transgenic *Arabidopsis thaliana* grown in soil. A**. Representative phenotype of the complement analysis of 14-day-old Control (pCambia 1302-GFP), MxIRT1, and MxIRT1 lysine site-directed mutagenesis (K165R-GFP, K196R-GFP, K165/196 R-GFP) transgenic *Arabidopsis thaliana* seedlings grown in soil. Scale bars = 1 cm. **B**. Leaf length and leaf width of the 14-day-old transgenic *Arabidopsis thaliana* in soil. Every independent measurement contains three replicates. Bars, mean ± standard errors (n = 3). Different letters indicate significant differences (P < .05) as determined by ANOVA. **C**. Immunoblot detects the expression of the Control, MxIRT1, and lysine point-directed mutants using GFP antibody in transgenic *Arabidopsis thaliana*. **D**. Fe concentration of Control, MxIRT1 and lysine site-directed mutagenesis transgenic *Arabidopsis thaliana* seedlings grown in soil using ICP-MS. Every independent measurement contains 3 replicates. Bars, mean ± standard errors (n = 3). Different letters indicate significant differences (P < .05) as determined by ANOVA.

To confirm the role of K165 and K196 mediated iron information transfer process in iron homeostasis in plants, we constructed the stable Control (pCambia 1302-GFP), as well as the K165R, K196R, and K165/K196R transgenic Arabidopsis plants and treated them with different iron condition (-Fe, 1/2 MS, ++Fe). Complement analysis showed that K165R, K196R, and K165/K196R could rescue the growth of Arabidopsis mutant *irt1* (Figure 6a) and displayed larger leaf length and width than *irt1* (Figure 6b) grown in soil. Immunoblot analysis was used to confirm the protein expression of the Control, MxIRT1-GFP, K165R-GFP, K196R-GFP, and K165/K196R-GFP in transgenic Arabidopsis seedlings using a GFP antibody (Figure 6c). In addition, the iron concentration in the K165/K196R transgenic Arabidopsis seedlings was higher than that of the Control, MxIRT1, K165R, and K196R (Figure 6d), and similar to that of K165/K196R in yeast. Thus, impairment of K165 and K196 mediated monoubiquitination could improve the iron uptake ability of MxIRT1 in Arabidopsis.

**Figure 7.**
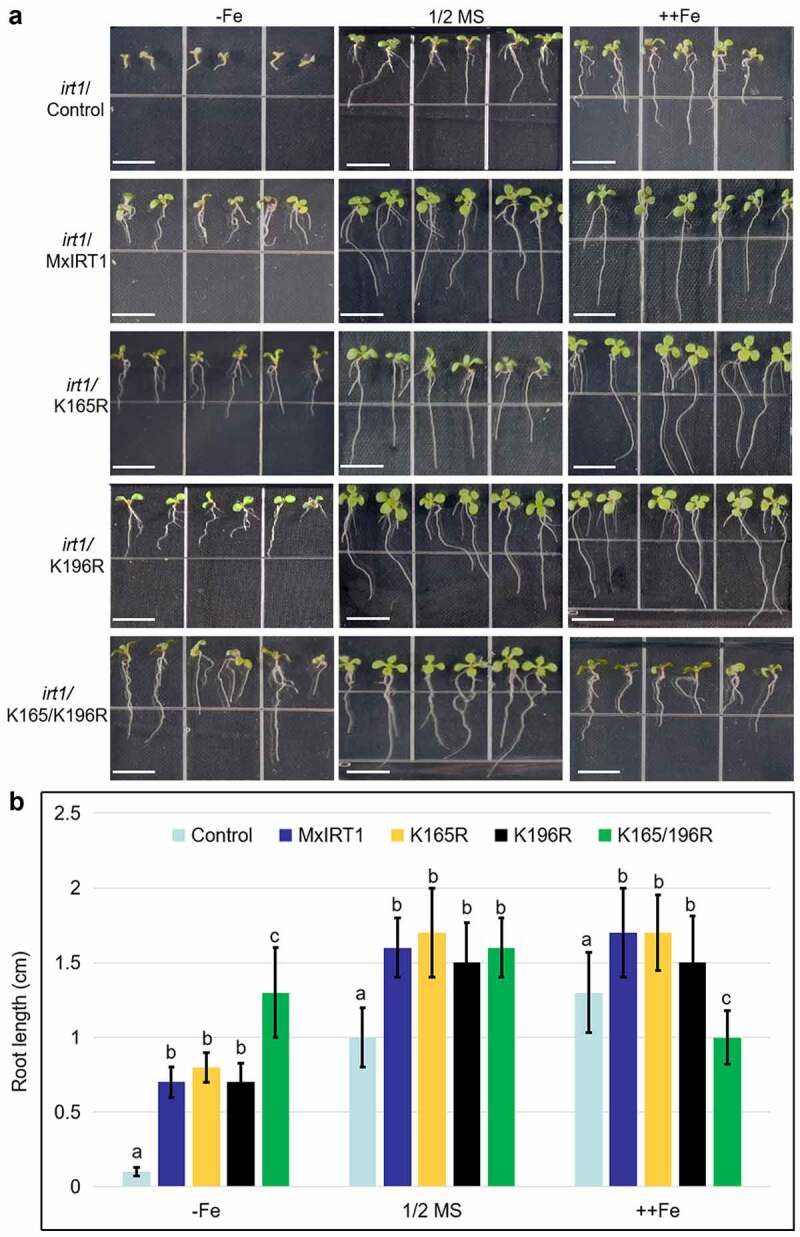
**Phenotypic analysis of lysine site-directed mutagenesis transgenic *Arabidopsis thaliana* under different iron conditions. A**. The representative phenotype of 7-day-old Control (pCambia 1302-GFP), MxIRT1 and lysine site-directed mutagenesis (K165R-GFP, K196R-GFP, K165/196 R-GFP) transgenic *Arabidopsis thaliana* grown in 1/2 MS medium, or 1/2 MS medium containing 300 µM Ferrozine (-Fe) or 1 mM EDTA-Fe (++Fe). Scale bars = 1 cm. **B**. Root length of Control, MxIRT1 and lysine site-directed mutagenesis transgenic *Arabidopsis thaliana* grown in 1/2 MS medium, or 1/2 MS medium containing 300 µM Ferrozine (-Fe) or 1 mM EDTA-Fe (++Fe). Every independent measurement contains three replicates. Bars, mean ± standard errors (n = 3); Different letters indicate significant differences (P < .05) as determined by ANOVA.

Subsequently, we detected the response of transgenic Arabidopsis seedlings to different iron conditions (Figure 7a). Under iron-deficient conditions, MxIRT1, K165R, K196R, and K165/K196R showed longer root lengths than the Control, with K165/K196A displaying the longest root length (Figure 7b). Under sufficient iron conditions, MxIRT1, K165R, K196R, and K165/K196R displayed the same root length. However, K165/K196A showed the shortest roots length compared to other transgenic Arabidopsis seedlings under excessive iron conditions. The retarded growth of K165/K196R transgenic Arabidopsis in excessive iron conditions might be caused by disrupting iron homeostasis in plants and iron toxicity after the inactivation of K165 and K196 mediated monoubiquitination. These results indicate that the mUb-MxIRT1 mediated iron information transfer process is necessary for maintaining iron homeostasis through not only regulating MxIRT1 endocytosis and retrograde transport for exocytosis in response to iron abundance, but also regulating MxIRT1 endocytosis and vacuolar degradation for turnover to avoid iron toxicity in response to iron excessive after iron sensing by HRM.

## Discussion

Plant cells often employ ubiquitination to enable rapid and dynamic signaling to maintain iron homeostasis by activating E3 ligases, deubiquitinating enzymes (DUBs), or Ub-binding proteins.^25^ COP9 subunit 6 (CSN6) downregulation in the early stage of iron deficiency leads to decreasing of IRON DEFICIENCY-RESPONSIVE ELEMENT-BINDING FACTOR 1 (IDEF) degradation and accumulation of IDEF1 protein, which up-regulates several iron uptake/utilization-related genes.^27^ Degradation of the transcription factor FIT is promoted by the E3 Ub ligases BRUTUS LIKE 1 (BTSL1) and BTSL2 activated by iron to prevent iron excess when plants transition from low to excess iron.^22^ In addition to the post-transcriptional regulation of key transcription factors to maintain iron homeostasis, the function and intracellular traffic of IRT1 could also be regulated at a posttranslational level to reply to metal change. AtIRT1 could bind directly with non-iron metals, leading to gradual removal from the PM by monoubiquitination on lysine residues K154 and K179 of AtIRT1, and was regarded as a bifunctional transporter-receptor.^3^ Therefore, research on the direct control of IRT1 ubiquitination and its localization and function by divalent metal, especially iron, has drawn much attention, and IRT1 could be established as one of the key models for studying the response of transporters to substrate change directly. In this study, we found that the monoubiquitination sites in MxIRT1, specifically K165 and K196 from the large loop, regulate the monoubiquitination-dependent endocytosis of MxIRT1, determining following vacuole degradation or retro-traffic for endocytosis to maintain iron homeostasis. In our previous studies, we found that the histidine-rich motif (HRM) in the same large loop acted as an iron sensor through the direct interaction of H192, a site specific to MxIRT1, with iron and regulated delivery of MxIRT1 vesicles to the PM after binding with iron.^30^ These results strengthen our faith that MxIRT1 is indeed the iron sensor. The K165 and K196-mediated monoubiquitination of MxIRT1 (mUb-MxIRT1) may act as a receptor involved in the iron information transfer process from the signal of the MxIRT1-Fe^2+^ complex to determine subsequent cellular traffic. Different iron statuses may change the structure of the MxIRT1-Fe^2+^ complex; after that, MxIRT1 in the PM transfers the signal of structure change of MxIRT1-Fe^2+^ to downstream effectors such as clathrin governed by monoubiquitination. The structure change of MxIRT1-Fe^2+^ complex in different iron conditions may need further research. Moreover, the endocytosis deficient mutant K165/K196R could increase tolerance to iron starvation (Figure 7a) and accumulate higher iron concentration (Figure 6d) in transgenic yeast and Arabidopsis. As over-expression of MxIRT1 has been reported to increase iron and zinc content in T3 transgenic rice seeds, and it was regarded as a good candidate gene for plant Fe and Zn biofortification in our previous work,^26^ the double lysine site-directed mutant K165/K196R could be exploited for biofortification of crops, such as rice whose edible portion of the seed has poor microelements.

In addition to K165 and K196, we found that K26, a site in the uncleaved signal peptide (SP) of MxIRT1, is also involved in MxIRT1 ubiquitination. Replacement of K26 (K26R) resulted in losing the ability of metal transport and PM targeting (Figure 4), and K26R was mainly co-localized with the ER marker Sec24, indicating that K26 was necessary for ER export. There is a novel folding assistance system that operates on membrane proteins from the cytosolic side of the ER.^20^ Folding of the Wnt signaling coreceptor LDL Receptor-related Protein 6 (LRP6) is promoted by monoubiquitination of a specific lysine, retaining it in the ER while avoiding degradation. Subsequent ER exit requires the removal of ubiquitin from this lysine by the deubiquitinating enzyme USP19.^20^ Considering K26 in uncleaved SP could be monoubiquitinated and it is necessary for the ER exporting function in MxIRT1, the K26-mediated ubiquitination may be required for MxIRT1 normal folding in ER through inhibiting the cleavage of SP from MxIRT1 and maintaining correct transmembrane domain formation, for iron sensing and PM fusion.

Many studies have uncovered novel factors involved in IRT1 recycling to the PM and facilitated a better understanding of the role of this intracellular traffic in the fine balancing of plant metal homeostasis. Elevated concentrations of non-iron metal allow the recruitment of the CBL-INTERACTING PROTEIN KINASE 23 (CIPK23) kinase and the subsequent phosphorylation of IRT1 at serine and threonine residues in this large cytosolic loop, which creates a docking site for the RING E3 ligase IDF1, leading to the decoration of IRT1 with K63-linked Ub chains.^11^ Whether MxIRT1 could also be decorated with poly-ub chains or not and which factors regulate the monoubiquitination and polyubiquitination for MxIRT1 may need further research, especially the factors regulating mUb-MxIRT1 recycle back to the PM. In addition, whether MxIRT1 drives other aspects of plant metal responses after sensing iron change directly, such as genomic reprogramming, non-iron metal absorption, or hormone synthesis when iron availability changes, will also have to be investigated.

Altogether, after K26 mediated ubiquitination correctly directs MxIRT1 ER exporting for the PM secretory pathway, MxIRT1 vesicles fusion with PM in supplement with iron, K165 and K196 regulate monoubiquitination of MxIRT1 (mUb-MxIRT1), which could act as receptor transferring the iron status signal from HRM to downstream effectors to maintain iron homeostasis through clathrin-dependent endocytosis in iron sufficient condition, docking closed to PM for decreasing iron availability or targeting to vacuolar degradation after endocytosis in excessive iron condition.

## Supplementary Material

Supplemental Material
