# Supplementary material for: Monoubiquitinated MxIRT1 acts as an iron receptor to determine MxIRT1 vacuole degradation or plasma membrane recycling via endocytosis
Source: Plant Signal Behav. 2022 Jul 1;17(1):2095141. doi: 10.1080/15592324.2022.2095141 (PMC9255258; doi:10.1080/15592324.2022.2095141)

**Supplemental Tables**

**Table S1. Primer names and sequences**

**Supplemental Figures**

**Figure S1. Numbers of cells having MxIRT1-GFP or Fet4-GFP in the PM every one hundred protein-expressing cells under different iron conditions.** Every independent measurement contains three replicates. Bars, mean ± standard errors (n = 3). Different letters indicate significant differences (P<0.05) as determined by ANOVA.

**Figure S2 Numbers of cells having K26R-GFP only in ER or colocalization with PM maker (in PM) every one hundred K26R-GFP expressing cell.** Every independent measurement contains three replicates. Bars, mean ± standard errors (n = 3). Different letters indicate significant differences (P<0.05) as determined by ANOVA.

**Table S1. Primer names and sequences**

| Primer name | Primer sequence |
| --- | --- |
| MxIRT1-F | 5’-CTTCCCTCTTGTCACTCGTT-3’ |
| MxIRT1-R | 5’-TATCATTGTCCATTCAGTTGTTAT-3’ |
| K26R-F | 5’-TCACCCCCAGAGCTTATTCA-3‘ |
| K26R-R | 5’-TGAATAAGCTCTGGGGGTGT-5’ |
| K165-F | 5’-TTATCCCAGATAAAGGTGAAACTC-3’ |
| K165-R | 5’-GAGTTTCACCTTTATCTGGGATAA-3’ |
| K196R-F | 5’-GTTGACAAAGGAGAAAACGGAG-3’ |
| K196R-R | 5’-CTCCGTTTTCTCCTTTGTCAAC-3’ |

**Figure S1. Numbers of cells having MxIRT1-GFP or Fet4-GFP in the PM every one hundred protein-expressing cells under different iron conditions.** Every independent measurement contains three replicates. Bars, mean ± standard errors (n = 3). Different letters indicate significant differences (P<0.05) as determined by ANOVA.


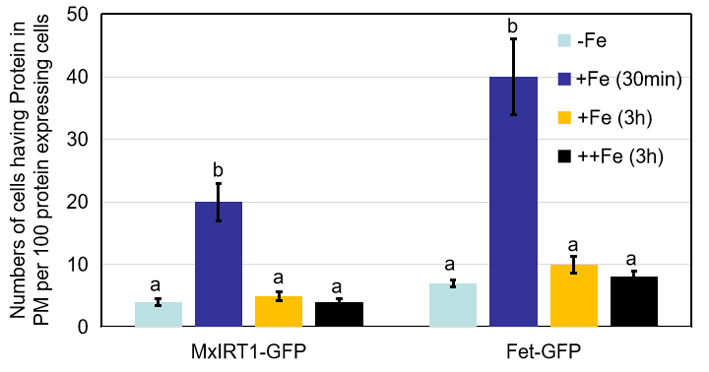


**Figure S2 Numbers of cells having K26R-GFP only in ER or colocalization with PM maker (in PM) every one hundred K26R-GFP expressing cell.** Every independent measurement contains three replicates. Bars, mean ± standard errors (n = 3). Different letters indicate significant differences (P<0.05) as determined by ANOVA.


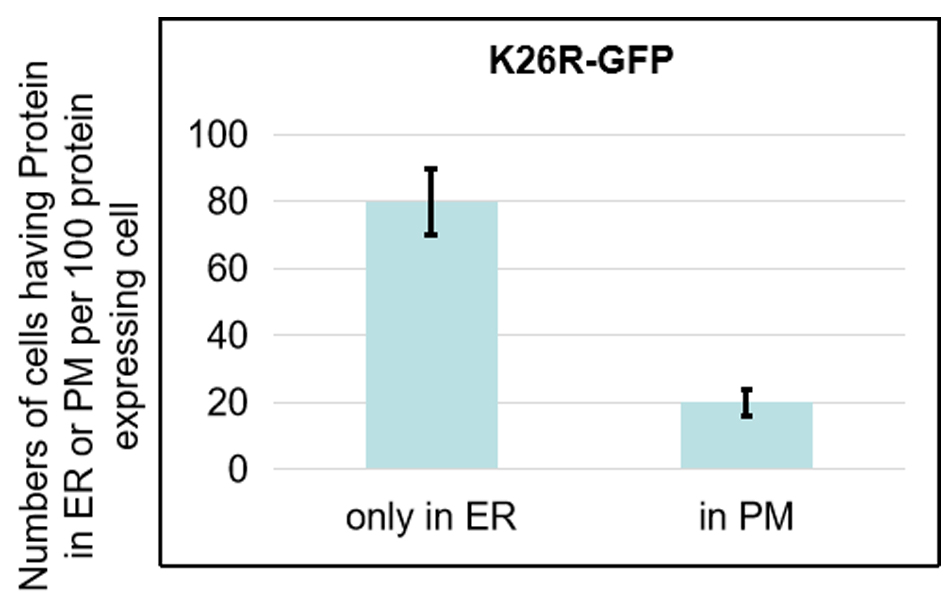

Supplement: Supplemental Material [file KPSB_A_2095141_SM2685.doc]
